# Structure-activity relationship of captopril derivatives as New Delhi metallo beta-lactamase 1 inhibitors

**DOI:** 10.5599/admet.3304

**Published:** 2026-05-24

**Authors:** Ikhlas Jarrar

**Affiliations:** Department of Pharmaceutical Chemistry, Faculty of Pharmacy, Arab American University, Jenin, Palestine

**Keywords:** Zinc binding group, fluorinated analogues, stereochemical effects, pyrrolidine ring, mercaptopropionamide, assay heterogenicity

## Abstract

**Background and purpose:**

New Delhi Metallo beta-lactamase-1 (NDM-1) is a zinc-dependent enzyme that confers resistance to several antibiotics; therefore, there is an urgent requirement for effective inhibitors. Captopril has been exploited as a scaffold in the design of NDM-1 inhibitors; however, a comparative evaluation of these derivatives from structure activity relationship perspective has not been conducted. This review aimed to evaluate captopril-derived NDM-1 inhibitors and to identify possible structure-activity relationships that govern their NDM-1 inhibitory action.

**Experimental approach:**

The literature was searched in a structured manner using scholarly databases to locate original studies that reported captopril derivatives and evaluated them in vitro against NDM-1 with the explicit reporting of the inhibitory concentration values (IC_50_). Eligible studies were filtered using predefined criteria and analysed using a qualitative approach, because heterogeneity in assay conditions and experimental methods prevented a direct quantitative comparison across studies.

**Important findings:**

The activity of captopril derivatives depends on the free thiol group (masking it reduces activity), with stereochemistry governing optimal binding orientation within the NDM-1 active site, hydrophobic substitutions enhance activity only within steric limits, and the carboxylate motif serves as a secondary anchoring feature.

**Conclusion:**

Captopril emerges as a promising scaffold for NDM-1 inhibitors and reveals significant structural features associated with NDM-1 inhibitors. Despite limited in vivo data and heterogeneity in assay conditions, the findings provide a rational framework for optimizing captopril-inspired NDM-1 inhibitors.

## Introduction

New Delhi metallo beta-lactamase-1 (NDM-1) is a major contributor to the global rise of antimicrobial resistance due to its ability to hydrolyse most beta-lactam antibacterial agents, including last-line carbapenems [1]. NDM-1 was first identified in 2008 in *Klebsiella pneumoniae* isolate from a Swedish patient previously hospitalized in India [2]. Since then, NDM-1 has emerged as a significant antimicrobial resistance mechanism [3] and has been detected in *Escherichia coli*, *Proteae*, and *Acinetobacter species* and other *Enterobacterial species* [4-7]. NDM-1 is often encoded in gene cassettes within integrons and plasmids, along with other resistance genes, thereby promoting its spread through horizontal gene transfer [8].

NDM-1 belongs to the B1 subclass within the metallo-beta-Lactamase (MBLs) and is a zinc-dependent enzyme in which catalysis is driven by a metal-activated hydroxide ion that promotes β-lactam ring hydrolysis. Consequently, NDM-1 is resistant to traditional serine lactamase inhibitors such as sulbactam, clavulanic acid and avibactam. Interestingly, monobactams are stable against MBL due to their monocyclic ring, which maintains spatial separation from the zinc ion in the active site [4,8-10].

NDM-1-producing bacterial strains exhibit resistance to a wide variety of antibacterial agents, including carbapenems, which are a last-resort therapy for multidrug-resistant bacterial infections. NDM-1 has received special focus because of its wide substrate coverage, high rate of spread through mobile genetic elements and high mortality rates associated with infections caused by NDM-1-producing bacteria. Moreover, these strains spread quickly within and across species and they have been detected in both drinking water and wastewater, highlighting the complexity of combating their spread [11-13].

The development of novel antibacterial agents that are naturally resistant to hydrolysis by NDM-1 is a complex, time-consuming, and expensive process, highlighting the importance of an alternative approach, such as restoring antibiotic potency by using inhibitors that shield β-lactams against NDM-1 hydrolysis. To date, there is no clinically approved inhibitor for NDM-1, despite extensive *in silico* and *in vitro* studies [1,4,11]. The variability in the entry loop permutation of the active site, the limited number of scaffolds capable of selectively targeting the NDM-1 active site, and the presence of multiple NDM-1 variants are all factors that impede the development of effective NDM-1 inhibitors [4,11,14].

NDM-1 inhibitors are commonly classified as covalent and noncovalent. Noncovalent inhibitors include zinc-binding thiols, bicyclic boronate (taniborbactam) and metal chelating agents, while compounds such as nitroprusside, p-chloromercuribenzoate (p-CMB), cefaclor and ebselen are among the covalent ones [14-24].

From a medicinal chemistry perspective, various scaffolds have been explored for NDM-1 inhibition, including metal chelating agents, cyclic boronate scaffolds and thiol-based scaffolds like captopril and related sulfuric derivatives (carboxymethyl mercaptoacetate thioether, mercaptopropionic acid and ebsulfur). Captopril is a biologically validated scaffold. Although captopril itself is not a clinically suitable NDM-1 inhibitor, crystallographic and medicinal chemistry studies have shown that it provides an effective starting point for optimization [11,25-30,38-43].

In the last decade, several medicinal chemistry studies have investigated captopril-based and captopril-inspired derivatives to enhance potency, selectivity, and binding affinity toward MBLs and NDM-1, specifically. These studies demonstrate the usefulness of the captopril scaffold in the rational design of inhibitors targeting MBLs, NDM-1 and DapE [16,20-22,31-43]. Although an increasing number of studies have been conducted, a systematic synthesis of the existing structure-activity relationship information on captopril-derived NDM-1 inhibitors remains lacking. Thus, the current paper serves as a review and comparative analysis of captopril-based inhibitors, particularly regarding trends in IC50 values and structural characteristics that influence NDM-1 inhibitory activity, and it is anticipated that the findings from this study will facilitate the discovery of NDM-1 inhibitors by providing guidelines for further lead optimization.

## Literature search and data collection

This review was conducted using a structured literature search and predefined inclusion criteria to identify studies reporting IC_50_ values for captopril-derived NDM-1 inhibitors. Studies were included if they met all the following criteria: 1) Biological inhibitory activity assays were performed on NDM-1; 2) The compounds investigated were clearly mentioned in the title or abstract as captopril derivatives, captopril-inspired derivatives or captopril-based derivatives; 3) The inhibitory concentrations (IC_50_) of captopril derivatives are reported clearly; 4) Original peer-reviewed scholarly articles that are published in English. Studies were excluded if they met any of the following criteria: 1) Biological inhibitory activity assays were not performed on NDM-1 specifically; 2) Reviews, editorials, or mechanistic studies without IC_50_ data.

The search was performed across PubMed, Google Scholar and Europe PMC and included literature published until February 2026. The search strategy combined free-text terms of interest, including the biological target and the inhibitor scaffold, using Boolean operators (AND/OR). The key search terms were NDM-1, New Delhi metallo-beta-lactamase-1, metallo-beta-lactamase, captopril, captopril derivatives, and captopril analogues. The search strategy was modified to suit the syntax and search interfaces of the respective databases. Titles and abstracts were screened to identify relevant studies, followed by full-text evaluation based on the inclusion criteria. Study selection and data extraction were performed by a single reviewer (the author).

**Figure 1.**
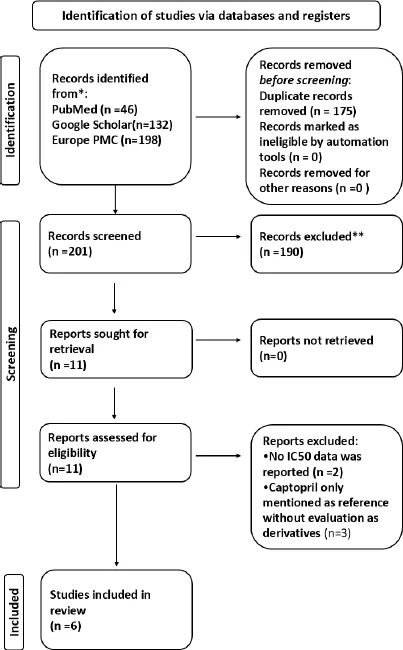
Flow diagram of the literature search and study selection process for studies included in the review

The database search revealed a total of 376 records: 46 in PubMed, 132 in Google Scholar and 198 in Europe PMC. After eliminating 175 duplicate records, 201 unique records remained for screening titles and abstracts. Of these, 190 were eliminated as apparently irrelevant. The remaining eleven reports were retrieved. and were screened to determine eligibility. Following full-text assessment, five articles were excluded. Finally, six studies were found to fit the inclusion criteria and have been incorporated in the review for systematic SAR analysis. The study selection process is summarized in Figure 1.

## Captopril scaffold and key pharmacophoric features

**Figure 2.**
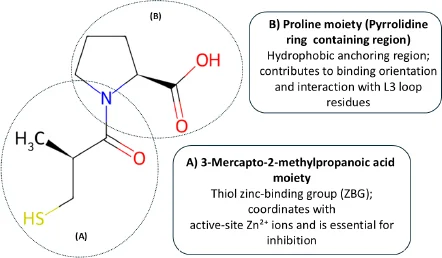
Chemical structure of captopril, highlighting key pharmacophoric features

Captopril is an angiotensin converting enzyme inhibitor widely used as an antihypertensive agent and has been repurposed as a starting scaffold for the design of NDM-1 inhibitors [16,30-43]. Captopril is an experimentally validated inhibitor of NDM-1 as it can interact with the active site via metal coordination. Crystallographic evidence confirmed that captopril binds in the NDM-1 active site, supporting its role as a lead compound for inhibitor design [26,39]. The inhibitory activity of captopril is based on a defined pharmacophore composed of a 3-mercapto-2-methylpropanoyl fragment attached to a proline moiety, as shown in Figure 2.

**Figure 3.**
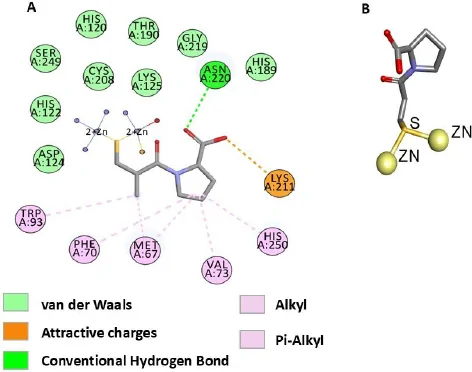
A: Two-dimensional interaction map of captopril within the NDM-1 active site, highlighting dual zinc coordination, hydrogen bonding, electrostatic and hydrophobic interactions; B: Corresponding three-dimensional structure demonstrating thiol-mediated coordination. Figure is generated by the author using BIOVIA Discovery Studio based on publicly available Protein Data Bank data (PDB ID: 5ZJ2) [38]

The thiol group is the main anchor, which coordinates the binuclear Zn (II) centre and, therefore, disrupts the catalytic activity, while the proline moiety prevents conformational flexibility and promotes a binding geometry favourable for enzyme inhibition [36-39]. Simplification studies without altering the thiol group have produced more active analogues with low-micromolar IC_50_ values [40]. Stereochemistry plays a significant role in the activity of captopril. D-captopril has been shown to be significantly more potent against NDM-1 than L-captopril in the same assay format, with reported IC_50_ values of 20.1±1.5 and 157.4±1.3 μM, respectively [39], whereas previously measured values were 7.9 μM for D-captopril and 39 μM for L-captopril, demonstrating the same pattern [40], even though absolute potency varied according to assay conditions. Such stereochemical effects were attributed to variation in the binding orientation and geometry of metal coordination in the active site of NDM-1, where the D-configuration places the thiol group in a favourable position within the NDM-1 active site [39]. For both captopril structures, the pyrrolidine ring of the proline moiety participates in hydrophobic interactions with valine (V73) from the L3 loop, whereas the carboxylate group is involved in a hydrogen bond with the backbone amine of asparagine (N220). D-captopril adopts an altered binding orientation to maximize interactions with the enzyme by increasing hydrophobic interactions with methionine (M67) and phenylalanine (F70) residues. Additionally, the carboxylate group of D-captopril forms water-mediated hydrogen-bonding interactions with lysine (K211), leucine (L218), and histidine (H189) residues. The methyl group of captopril is also involved in the interaction with the enzyme through hydrophobic interactions with methionine (M67) and tryptophan (W93) [39], as shown in Figure 3.

## Assay heterogeneity and IC_50_ determination

The included studies used various assays to determine IC_50_ values for NDM-1 inhibition. Methodological variability in substrate selection, detection methods, and reaction conditions was observed across studies. These differences influence inhibitory potency and limit the reliability of direct numerical comparisons across studies. Therefore, cross-study comparisons were performed conservatively and treated as qualitative unless the same assay formats were used. To address this variability, the included studies were classified according to the primary assay used to determine IC_50_ values to support within-group comparability, as shown in Table 1.

**Table 1.** Summary of IC_50_ assays and robustness across NDM-1 inhibitor studies

Study group	Study	Primary IC_50_ assay	Secondary validation assay (orthogonal, selectivity, safety)	IC_50_ readout	Methodological robustness of IC_50_ reporting	Limitation
Group A	[41]	Fluorogenic kinetic hydrolysis (fluorocillin)	MIC determination (broth microdilution, *K. pneumoniae E. coli*); FICI synergy analysis; cytotoxicity assay (CCK-8 assay, LDH release, *in vivo* acute toxicity in mice)	Fluorescence kinetics (RFU/time)	High	ACE activity is not assessed; Limited mechanistic depth
	[42]	Fluorogenic kinetic hydrolysis (fluorocillin)	Spectrophotometric imipenem hydrolysis assay and Ki determination; ACE selectivity; MIC potentiation assays (imipenem +tested compound); Determination of binding pose via molecular docking and dynamic studies	Fluorescence kinetics (RFU/time)	High	Human cell viability and cytotoxicity are not assessed
Group B	[43]	Spectrophotometric assay of meropenem with initial rate IC_50_ determination	MIC potentiation assays (meropenem +tested compound); Prediction of binding pose via solution state NMR and molecular docking studies	absorbance at 360 nm (meropenem hydrolysis)	High	ACE activity is not assessed; Human cell viability and cytotoxicity are not assessed
	[38]	Spectrophotometric assay of imipenem with initial rate IC_50_ determination	MIC potentiation assays (meropenem +tested compound)		High	
Group C	[39]	Kinetic-based inhibition assay*	MIC determination (broth microdilution, E. coli); X-ray crystallography (NDM-1); ACE activity is assessed	Not mentioned clearly*	High	Limited mechanistic depth, primary IC_50_ assay is not mentioned clearly*
	[40]	Prescreen (inhibition, % at 1 mM) → dose response IC_50_ determination	MIC determination (broth microdilution, K. pneumoniae E. coli); X-ray crystallography (BcII/IMP-1/VIM-2 captopril complexes not NDM-1)	Not reported in the context**	Low	Assay conditions not fully reported; limited mechanistic depth; no orthogonal validation; ACE activity is not assessed; human cell viability and cytotoxicity are not assessed

*Primary IC_50_ assay is referenced to prior methods [44,45]

**Cell based testing and co-crystallization with NDM-1 were stated to be ongoing, but no results were reported in the study

Group A comprised fluorogenic kinetic hydrolysis assays, whereas group B included studies using spectrophotometric hydrolysis assays. Group C encompassed two studies that did not fit into either approach.

Methodological robustness was further evaluated based on the presence of supporting evidence beyond the primary assay. Orthogonal validation refers to functional assays that confirm IC_50_ using different experimental formats such as minimum inhibitory concentration (MIC) assays, antibiotic potentiation assays, FICI analysis, or kinetic inhibition assays. Safety evaluation refers to cytotoxicity and *in vivo* toxicity testing.

Selectivity assays are experiments used to assess activity against angiotensin-converting enzymes (ACE), helping determine whether compounds retain specificity for NDM-1 while minimizing potential off-target effects associated with the captopril scaffold. These assays are important for evaluating therapeutic safety and distinguishing optimized derivatives from non-selective inhibitors. Structural and biophysical characterization involves X-ray crystallography and NMR, which determine the binding interactions between the inhibitor and NDM-1.

Robustness was considered high when the IC_50_ was supported by at least one orthogonal secondary assay confirming inhibitor activity in a different assay format. A Moderate level of robustness was assigned when secondary evidence was present, without orthogonal validation to confirm the results from the primary IC_50_ assay. However, the studies may still include additional safety, selectivity, and structural assays, such as X-ray crystallography, molecular docking, or dynamics studies. A low level of robustness was assigned when conclusions relied primarily on the primary IC_50_ essay, with no secondary evidence. Notably, structural or computational data alone, without orthogonal validation, were insufficient to achieve high robustness, as they did not directly confirm inhibitory activity.

## Structure-activity relationships across groups

### Thiol as a zinc-binding group and thiol masking

All study groups identified the free-thiol zinc-binding group (ZBG) as an important driver of NDM-1 inhibition [38-43]. Among group A, Meng *et al.* [41] retained the free thiol moiety and demonstrated compounds like compound **14a** (IC_50_ = 0.1 μM) with enhanced potency. This study also provided direct structural support by solving high-resolution NDM-1 complexes with captopril and its optimized derivatives, thereby linking potency changes to specific binding interactions [41]. Similarly, Alfano *et al.* [42] demonstrated that retaining the free thiol moiety among series **6a-f/7a-d** maintained activity, whereas thioester-protected analogues in series **14** were poorly active.

In group B, scaffold optimization strategies consistently preserved the free thiol group, further supporting its essential role in NDM-1 inhibition. Ma *et al.* [38] synthesized 10 compounds, six of which (**3**, **4**, **6**, **7**, **8**, **11**) were more potent than D-captopril. Additionally, among fluorinated analogues designed by Kondratieva *et al.* [43], preserved activity against NDM-1 was observed when the free thiol moiety was preserved, with only a minority showing loss of inhibition.

In group C, Li *et al.* [40] observed loss of inhibition upon thiol protection in simplified analogues, supporting a requirement for a free thiol group for activity under their assay conditions. Some analogues were highly potent, like compound **22** (IC_50_ approximately 1.5 μM), along with others that remained active [40]. Brem *et al.* [39] further provided structural evidence and additional inhibition data, showing that D-captopril (IC_50_ ≈20 μM) is more potent than L-captopril due to the orientation of the thiol group in the active site.

Detailed structures of all compounds discussed are presented in Supplementary Table S1.

Overall, the collective evidence across all study groups supports the conclusion that a free thiol is a required moiety for activity, whereas masking the thiol group impairs activity by disrupting zinc coordination within the NDM-1 active site.

### Stereochemistry

Stereochemistry was explored from various aspects across all study groups [38-43]. Among group A Meng *et al.* [41] separated selected racemic mixture of some compounds with promising activity into individual diastereomers and evaluated them independently, demonstrating that (R, S) configuration was more potent than the (S, S) configuration among all tested compounds, some potent compounds like compound **14m** and others showed that activity was enhanced with R configuration at the chiral carbon adjacent to thiol moiety. Additionally, Alfano *et al.* [42] reported that (S, R) configuration was more potent than (S, S) configuration, indicating that potency is increased with the R configuration, but here, instead of the chiral carbon adjacent to thiol, the R configuration is preserved at the carbon adjacent to nitrogen located in the indoline moiety.

In group B, Kondratieva *et al.* [43] further supported the importance of stereochemistry by identifying the fluorinated stereoisomer **(2R,2′R)-5αC** as the most important derivative in their series with (IC_50_ = 0.3 μM). Ma *et al.* [38] also addressed stereochemical influence indirectly, as they proposed that the most active compound was compound **11** (IC_50_ = 4.6 μM), which formed a racemate dimer that mimics the binding mode of hydrolysed beta lactam binding geometry, and co-crystal structural analysis highlighted how stereochemical geometry governs active site interaction.

In group C, Brem *et al.* [39] provided strong stereochemical evidence, demonstrating that D-captopril was the most active captopril stereoisomer against NDM-1 (IC_50_ = 20.1 μM) and its activity is reduced with epimerization (IC_50_ = 64.6 μM), whereas L-captopril was substantially weaker (IC_50_ = 157.4 μM) and epi-L-captopril showed nearly lost of activity (IC_50_ > 500 μM). Li *et al.* [40] similarly noted that in simplified free acid and benzylamide analogues, the R configuration at the thiol adjacent stereocenter was related to higher potency. Conversely, more polar derivatives, such as Weinreb amides, N-methoxy amides, the S configuration became more favourable, reflecting that more polar and flexible substituents needed a different geometry to maximize the interactions in the active site [40].

Overall, the collective evidence indicates that stereochemical effects are controlled by the immediate environment of the substituents, in which the best configuration represents a balance among zinc coordination, hydrogen bonding and steric fit, rather than an inherent R/S preference [38-43].

### Anchoring group effects

All the study groups reinforce the significant role of the carboxylate moiety as an anchoring group, either directly or indirectly [38-43]. In Group A, evidence was not extensive but favourable. Alfano *et al.* [42] replaced α-carboxylic acid with 2-carboxamide in their indoline **6a-d** series, then substituted it with a glycyl amide moiety in **7a-d** series. The findings demonstrated that compounds retaining the original carboxamide arrangement were more active (up to 3-fold more) than their corresponding 2-glycylamide analogues [42]. These findings suggest that the original carboxamide structure is beneficial to inhibition [42]. Within the same group, Meng *et al.* [41] preserved the carboxylic acid while converting the proline scaffold to other analogues throughout their series and did not vary the carboxylate motif or its orientation. They suggested that the carboxyl group is pivotal for NDM-1 inhibition because of its ability to form a hydrogen bond with Asn220 and should be maintained in the newly designed analogues [41].

More direct evidence is provided by Ma *et al.* [38], who clearly noted that the carboxylate position in D-captopril derivatives affect NDM-1 inhibition. For example, potency decreased after shifting the carboxylate moiety from the ortho position, as shown in compound **3** (IC_50_ = 6.9 μM) to the meta position and compound **5** (IC_50_ =28.4 μM), while relocation to the para position and compound **6** (IC_50_ 4.9 μM) improved activity [38]. These effects are supported by structural analysis, as the para carboxylate position strengthened hydrophobic interactions with F70 and formed two water-mediated hydrogen bonds stabilized by N220, K211, and H250, whereas the meta isomer weakened both hydrophobic and hydrogen-bonding interactions [38]. Moreover, substitution of the para-carboxylate with an amide decreased potency, as observed in compound **7** (IC_50_=11.1 μM), because hydrogen bonds to water molecules were weakened either by a longer distance or by a lack of stabilization [38]. Kondratieva *et al.* [43] also explored the significance of the carboxylic acid moiety by deleting it or changing its position and orientation, but its influence seems more context-dependent within the fluorinated series than uniformly dominant. In some analogues, carboxylate removal was tolerated, whereas in others it caused significant loss of activity, indicating context-specific contributions rather than absolute dominance [43].

In group C, Li *et al.* [40] also provided indirect support for the anchoring role of the carboxylate group in simplified captopril analogues. Compounds with a free carboxylic acid have been demonstrated to exhibit measurable NDM-1 inhibition, while masking the acid by methyl ester blocked the activity, as noted in compound **9** (IC_50_ > 200 μM) [40]. Notably, highly potent simplified analogues, such as compounds **21** and **22** (IC_50_ 5 ± 0.4 and 1.5 ± 0.2 μM, respectively), also retained the free carboxylate moiety, further reinforcing its compatibility with strong inhibition [40]. Also, Brem *et al.* [39] demonstrated that stereochemistry regulates the presentation of the carboxylate to the conserved basic residues within the active site, thereby providing a structural explanation for the superior potency of D-captopril over the L enantiomer

In general, all the study groups combined support that the carboxylate moiety is a major anchoring moiety in NDM-1 inhibitors and primarily through its role in hydrogen bonding, position optimization, and stabilization within the active site. Its specific contribution, however, can vary with the context of scaffolds, the orientation of substituents, and the overall structural environment.

### Pyrrolidine ring modification

Group A studies redesigned the pyrrolidine scaffold to assess the influence of ring geometry on activity, rather than directly modifying the pyrrolidine ring. Alfano *et al.* [42] replaced the pyrrolidine ring of captopril with an indoline bicyclic system, and Meng *et al.* [41] replaced the pyrrolidine ring with variant scaffolds including L-tryptophan, L-phenylalanine, L-homophenylalanine, biphenyl, indole, or 2-benzo[b] thiophene-derived frameworks. Both studies showed that replacement of the pyrrolidine ring was well tolerated [41,42].

Clearer evidence can be derived from group B studies for the role of the pyrrolidine ring scaffold [37,42]. Ma *et al.* [38] showed that ring expansion from a five-membered pyrrolidine ring of D-captopril to a six-membered ring improved NDM-1 inhibition as shown in compound **3** (IC_50_ = 6.9 μM) compared to D-captopril (IC_50_=21.8 μM), whereas further expansion to a seven-membered ring reduced potency as shown in compound **4** (IC_50_=20.4 μM). These findings indicate that over-expansion interferes with optimal positioning in the active site [38]. Kondratieva *et al.* [43] further emphasized the importance of ring geometry, stereochemical arrangement and positioning of substituents through pyrrolidine to piperidine expansion in fluorinated captopril analogues. They found that some compounds retained high activity as compound **5αE** (IC_50_ = 3.2 μM), whereas other analogues were much less active as compound **5βE (**IC_50_ >145 μM) [43].

In Group C, Li *et al.* [40] simplified the pyrrolidine ring with less polar amide substituents, such as linear aliphatic amides and benzyl amides. Substitution with aliphatic amide produced moderately active compounds (IC_50_ ≈13 to 20 μM) and bulky heteroaromatic analogs were usually weak. On the other hand, benzyl amide substitution as in compound **21** (IC_50_ = 5 μM) and compound **22** (IC_50_ = 1.5 μM) resulted in potent compounds [40].

These findings suggest that while the original pyrrolidine ring is not a structural requirement, scaffold modifications must preserve the spatial orientation of the thiol zinc-binding group and the anchoring functionality to maintain effective inhibition. Collectively, evidence across all groups supports that excessive expansion or improperly oriented substituents reduce potency, whereas para-oriented expansion of a six-membered ring may enhance activity.

### Hydrophobic cap and steric ceiling

Hydrophobic substitution was often associated with enhanced binding to lipophilic regions close to the NDM-1 active site; however, this effect diminished with steric bulk substituents or unfavourable orientation [38-43]. This effect was especially noticeable in Group A. Meng *et al.* [41] developed mercaptopropionamide analogues by adding various hydrophobic motifs on both sides of the mercaptopropionamide core and demonstrated substantial potency improvement with various aromatic hydrophobic groups, as illustrated by biphenyl-containing compound **14a** (IC_50_ = 0.10 μM), and indole analogue compound **14m** (IC_50_ = 0.12 μM) [41]. Conversely, extending the hydrophobic system a notch further was not always advantageous, as observed with larger polycyclic compounds such as phenanthrene, dibenzo[b,d]thiophene, and trifluoromethylbenzene. These results indicate that the steric capacity of the binding pocket is already reached [41]. Similarly, Alfano *et al.* [42] demonstrated that the moderate hydrophobic expansion in their indoline-based series significantly improved potency and increasing alkyl bulk at the 3-position enhanced activity through hydrophobic capping, with the 3,3-diethyl analogue **6d** (IC_50_=3.5 μM) representing one of the more active compounds in this series.

In group B, Ma *et al.* [38] investigated hydrophobic substitution by expanding the D-captopril scaffold weather by ring expansion or aryl extension and noticed that moderate hydrophobic enlargement improved activity as shown in their six-membered ring analogue (compound **3**, IC_50_ = 6.8 μM), which was more effective than D-captopril, while excessive enlargement or poor orientation reduced potency, as seen in compound **10** (IC_50_ > 300 μM) [38]. Kondratieva *et al.* [43] further explored hydrophobic substitution by varying the hydrophobic cap in trifluoro-methylated mercaptopropionamide analogues, demonstrating that pyrrolidine and piperidine-based hydrophobic motifs, such as **5αC** (IC_50_ = 0.3 to 4.5 μM), were more favourable than poorly oriented β-CF_3_ analogues, which were often weak or inactive (IC_50_ > 300). This suggests that hydrophobic substitution tends to improve potency only when paired with α-CF_3_ configuration [43].

Group C provided more indirect evidence. Li *et al.* [40] found that some benzyl amide analogues represented some of the most effective simplified analogues, such as compound **22** (IC_50_ = 1.5 μM), which is about a five-fold better inhibitor than D-captopril (IC_50_ = 7.9 μM). However, alteration of the benzyl ring was not tolerated; for example, meta-hydroxybenzyl analogue compound **23** retained moderate activity (IC_50_ ≈5 μM), whereas an ortho-hydroxy substitution, methyl substitution, or fluorine substitution resulted in inactive compounds (IC_50_ > 200 μM) [40]. The significance of hydrophobic interactions is supported by structural information presented by Brem *et al.* [39], rather than by investigating the potential of hydrophobic substitution in captopril-based inhibition.

Overall, the collective evidence suggests the following SAR rule: hydrophobic capping can enhance potency by improving pocket occupancy, but the benefit is limited by a steric ceiling and poor orientation [38-43].

### Fluorinated analogues

Various fluorination strategies were explored across study groups [40,41,43]. In group A, Meng *et al.* [41] introduced a trifluoromethyl-substituted phenyl moiety, generating compound **14q**, which exhibited moderate activity (IC_50_ = 3.90 μM).

In group B, Kondratieva *et al.* [43] reported a systematic fluorination approach in which they used trifluoromethyl (CF3)-substituted mercaptopropionamide scaffolds while maintaining the thiol group. Activity in their series was highly dependent on the location of fluorination: αCF_3_ analogues were more active than βCF_3_ analogues. As an example, **5αC**, **5αE**, and **5αF** showed IC_50_ values of 0.3 to 4.5 μM, while numerous βCF_3_ analogues were weak or inactive [43]. These findings suggest that fluorination may enhance potency by improving hydrophobic interactions and conformational stability, but this enhancement relies on the location of the fluorinated group and the scaffold architecture [43].

From group C, Li *et al.* [40] explored fluorination less extensively, and used the peripheral aryl fluorination instead of core scaffold modification; however, ortho-fluorobenzyl compound **26** was not active (IC_50_ > 200 μM), which suggested that the simple fluorine substitution of the benzyl cap was not conducive in this simplified scaffold.

### Scaffold simplification

Li *et al.* [40] from group C investigated simplified captopril analogues. They found that the pyrrolidine residue could be substituted with less reactive amide fragments of 3-mercapto-2-methylpropanoic acid without significantly affecting activity, indicating that a considerable reduction in scaffold complexity could be achieved without loss of activity. In their simplified series, a simple hydrophobic benzyl amide was particularly favourable, such as analogue **22**, which was the strongest compound (IC_50_ = 1.5 μM) in the whole series [40]. Substitution on the benzyl ring was, however, poorly tolerated, with the meta-hydroxybenzyl analogue **23** remaining active (IC_50_ = 5 μM) while the ortho-hydroxy, methyl and fluor substituted analogues of the benzyl ring were not active. Further simplification of the structure generated compounds **31** and **32** that inhibit NDM-1 (IC_50_ = 15 and 10.4 μM, respectively), indicating that the thiol-containing moiety comprises the core of the essential inhibitory unit, though the proline moiety and the hydrophobic cap could also be simplified, although not with a wide structural range [40].

## Integrated SAR interpretation and strength of evidence

Since IC_50_ values are condition dependent, cross-group comparisons were interpreted qualitatively, and quantitative ranking was restricted to groups within the same assay format. To support SAR interpretations across study groups, the strength of evidence was assigned using predefined criteria for reproducibility and methodological alignment, as described in Table 2.

**Table 2.** Strength of evidence assessment

Strength of evidence	Criteria
High	Trend was observed in both studies within the same assay group (A or B) and supported by at least one additional study from another group (including C)
Moderate-high	Trend was observed in studies within the same assay group (A or B) without additional support from additional studies across groups, or from three studies from different assay formats, or from a single study supported by direct structural support such as X ray-crystallography or NMR
Moderate	Trend was observed by at least two studies from different assay groups (A+B or A+C or B+C)
Low	Trend based on a single study without structural support

**Figure 4.**
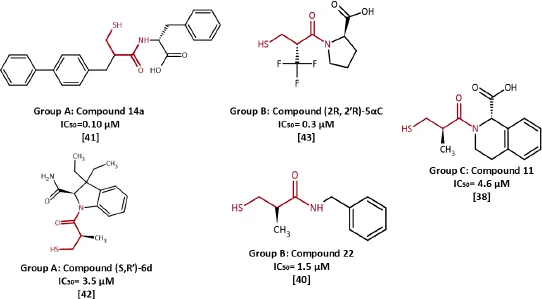
Representative structures of the most potent captopril derivatives by each study group (A-C). The conserved mercaptopropionamide zinc-binding region is shown in red. The IC_50_ values are reported as in the original studies

Evidence was ranked as high when the trends were reproduced in both studies within the same assay group and further supported by at least one additional study from another group. Moderate-High strength was assigned when trends were reproduced within a single assay group, with no supportive evidence from other groups. Moderate strengths were assigned when trends were observed across non-aligned studies without replication within the same assay group, and low strength of evidence when the trend was observed in a single study or in non-aligned studies. SAR features, direction, evidence source, and strength are shown in Table 3. The most potent compounds identified across included studies are summarized in Table 4, whereas Figure 4 shows their chemical structures.

**Table 3.** Structural features effects on activity and strength of evidence for captopril-inspired NDM-1 inhibitors

SAR feature	Effect on activity	Evidence strength	Evidence group & references	Comments
Free thiol zinc binding group (ZBG)	Essential for activity	High	A [41,42] B [38,43] C [39,40]	Required for Zn (II) coordination
Thiol masking	Decrease	Low	A [42]	Prevent Zn (II) coordination
Hydrophobic/steric substitution	Increase u*ntil steric ceiling*	High	A [41,42] B [38,43] C [40]	Pocket filling (until steric ceiling)
Pyrrolidine ring modification/ring scaffold geometry	Increase *replacement with indoline*	Low	A [42]	Excessive enlargements reduce hydrophobic contact; simplified scaffolds remain active; benzyl-ring substitutions (*e.g.* meta-methyl, ortho-F, ortho-OH) were weak, suggesting a constrained tolerance space
	Increase *replacement with benzyl amid*	Moderate-high	A [41] B [42] C [40]	
	Increase *ring expansion: 6-member ring* Decrease *ring expansion:* *7-member ring*	Moderate-high	B [38,43]	
Stereochemistry	D > L	Moderate-high	A [42] B [43] C [40]	Optimal carboxylate/proline placement in NDM-1 active site
	R > S *(at C adjacent to thiol)*	Moderate-high	A [41] B [43] C [40]	
	R > S *(at C adjacent to nitrogen)*	Moderate	A [42] B [43]	
α-carboxylate moiety on proline	Essential for binding	Moderate*	A [42] C [40]	Act as anchoring group in NDM-1 active site
Carboxylate position on the ring**	Para > ortho > > meta	Moderate-high	B [38] supported by structural evidence	Para carboxylate position strengthened hydrophobic interaction; meta carboxylate position weakened both hydrophobic and hydrogen bonding interactions
Carboxylate replacement carboxamide *vs.* glycylamide	Increase *carboxamide > > glycylamide*	Low	A [42]	Maintain an anionic/H-bonding anchor positioned for loop/water network interactions
Fluorination position (CF₃ placement: α *vs.* β)	α-CF₃ > β-CF₃	Low	B [43]	α-CF₃ oxidizes faster (stability issue)

*Although supported by X-ray crystallographic evidence, the α-carboxylate moiety is classified moderate due to the lack of direct substitution studies

**The ring is six membered

**Table 4.** The most potent captopril derivatives from each group (A-C) with their corresponding key modification

Group	Study	Number of synthesized derivatives	Compound	IC_50_, μM	*Key modification*
A	[41]	17	Compound 14a	0.1	Bulky aromatic
	[42]	13	Compound (S, R)-6d	3.5	Ring expansion: indoline scaffold
B	[43]	20	Compound (2R, 2′R)-5αC	0.3	Trifluoromethyl substitution
	[38]	10	Compound 11	4.6	Ring expansion: tetrahydroisoquinoline substituted ring
C	[40]	29	Compound 22	1.5	Simplified amide
	[39]	6	D-captopril	20.1	Core scaffold stereochemistry

## Limitation and future directions

Several limitations of this review should be addressed. Firstly, a small number of studies were included in this review, as only those that reported IC_50_ values against NDM-1 were considered, which may have excluded mechanistic studies of interest. Secondly, a high degree of assay heterogeneity in the covered studies, substrate selection and experimental conditions complicated the direct comparison of the inhibitory potency. As a result, the use of IC_50_ values should be approached with caution, as they are not fully standardized across studies. Moreover, most of the collected data are based on *in vitro* enzyme tests, with little in vivo or clinical confirmation. Another limitation worth mentioning is the inconsistent reporting of stereochemistry, as D/L nomenclature was not accompanied by R/S nomenclature. Lastly, since it is a single-author review, there may be inherent bias in the selection and interpretation process.

Future work must further explore optimization of captopril-derived scaffolds, ranging from balancing hydrophobic capping, fluorination, and thiol masking to enhance stability without compromising zinc-binding ability. Also, adopting consistent reporting with the R/S configuration is recommended to avoid confusion that can result from relying on D/L nomenclature alone. In addition, the assay conditions (*e.g.* substrates and zinc concentrations) also need to be standardized so that the IC_50_ values can be compared across studies more reliably. Moreover, clinical translation will be imperative, extending the studies beyond in vitro enzyme assays to in vivo efficacy, pharmacokinetics, and toxicity profiling. The binding interactions will be further elucidated through structural and mechanistic studies, especially by co-crystallization with NDM-1, and then used to design rationally. Lastly, the potential of these inhibitors as adjuvants when used alongside beta-lactam antibiotics should be thoroughly evaluated to address antibiotic resistance in clinical contexts.

## Conclusions

Antimicrobial resistance is one of the biggest challenges facing modern healthcare systems. β-lactamases, specifically NDM-1, significantly compromise the clinical efficacy of β-lactam antibiotics. To date, there are no clinically available NDM-1 inhibitors. Captopril has emerged as a validated lead scaffold to design novel derivatives, in particular, the activity is frequently retained in derivatives bearing the mercaptopropionamide motif, highlighting the essential role of the thiol-zinc binding group. Structural optimization strategies, including modification of ring geometry, enhancement of hydrophobic interactions, and fluorination, have demonstrated promising *in vitro* inhibitory activity. However, translation into clinically viable agents remains limited. The continued development of captopril-inspired derivatives will require better assay standardization alongside integrated structural optimization and biological validation to facilitate their advancement as effective NDM-1 inhibitors.

## Supplementary material


