# Supplementary material for: Structure-activity relationship of captopril derivatives as New Delhi metallo beta-lactamase 1 inhibitors
Source: ADMET DMPK. 2026 May 24;14:3304. doi: 10.5599/admet.3304 (PMC13499674; doi:10.5599/admet.3304)
Supplement: Supplementary file 1 [file ADMET-14-3304-S1.pdf]

ADMET & DMPK **14** (2026) S3304

Open Access : ISSN : 1848-7718
http://www.pub.iapchem.org/ojs/index.php/admet

Supplementary material to

Structure-activity relationship of captopril derivatives as New Delhi metallo beta-lactamase 1 inhibitors

Ikhlas Jarrar

Department of Pharmaceutical Chemistry, Faculty of Pharmacy, Arab American University, Jenin, Palestine

ADMET & DMPK **14** (2026) 3304; https://doi.org/10.5599/admet.3304

Table S1. Captopril derived NDM-1 inhibitors reported in the review, including compound names (Comp.), structures, inhibitory potency, effect on activity compared to D-captopril, and key structure-activity relationship (SAR) features

Group	Ref.	Compound	Structure	IC_{50}, µM	Activity	Activity compared to D-captopril	Key SAR features
A	[41]	**14a**	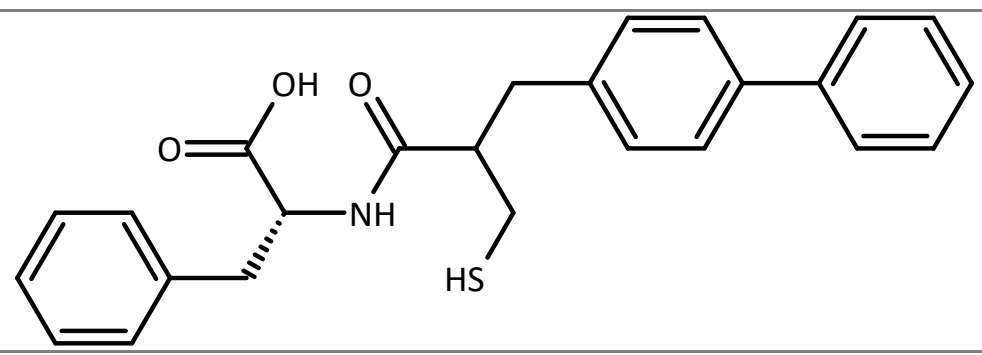	0.10	Very high	↑↑↑	Thiol (ZBG); Hydrophobic cap; Anchoring group effects; Pyrrolidine ring replacement
A	[41]	**14m-1 (S,S)**	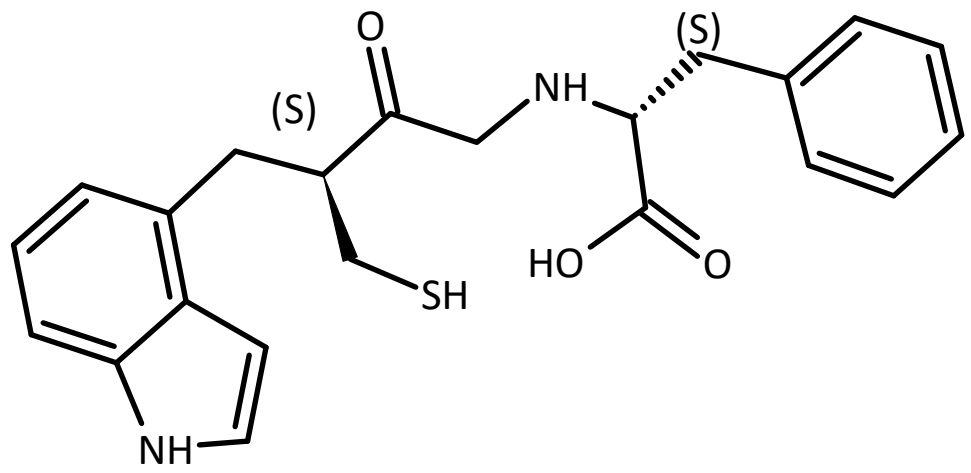	0.48	Very high	↑↑↑	Thiol (ZBG); Stereochemistry; Hydrophobic cap; Anchoring group effects; Pyrrolidine ring replacement
A	[41]	**14m-2 (R,S)**	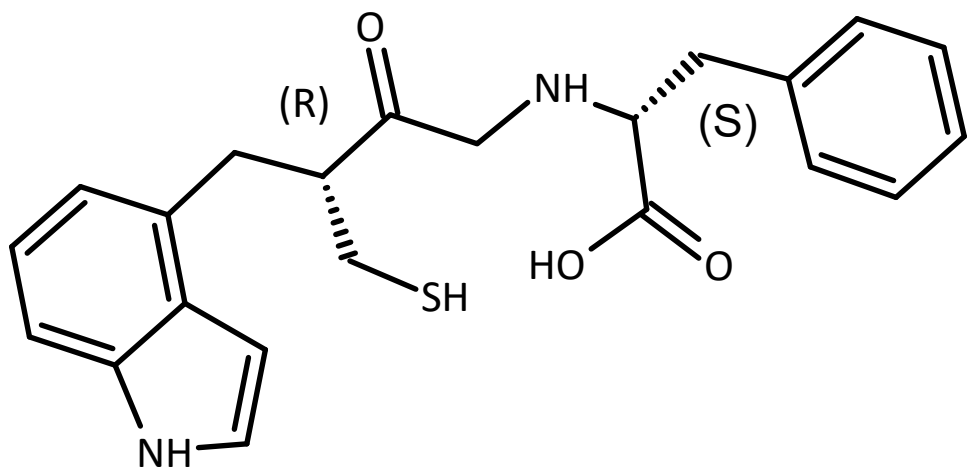	0.17	Very high	↑↑↑	Thiol (ZBG); Stereochemistry; Hydrophobic cap; Anchoring group effects; Pyrrolidine ring replacement
A	[41]	**14q**	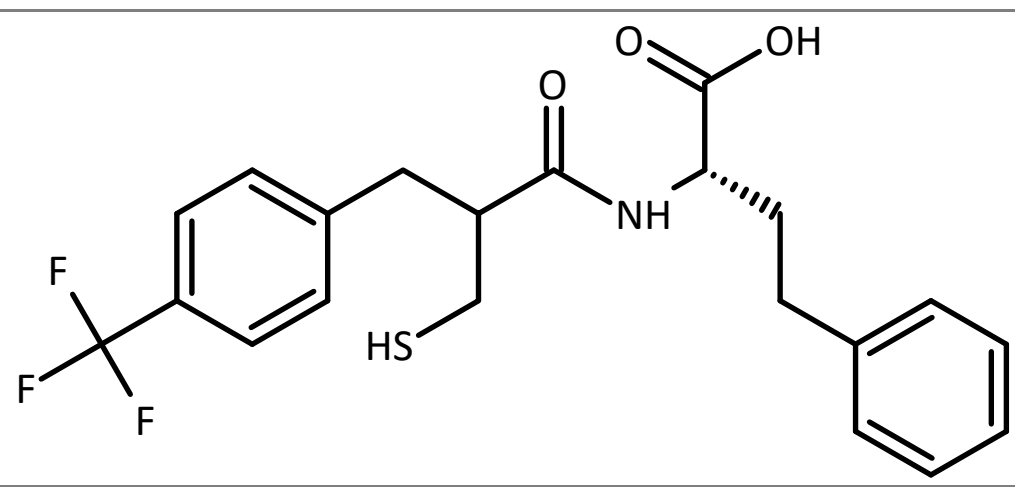	3.90	High	↑↑	Thiol (ZBG); Fluorination; Pyrrolidine ring replacement
A	[42]	**6a-d**	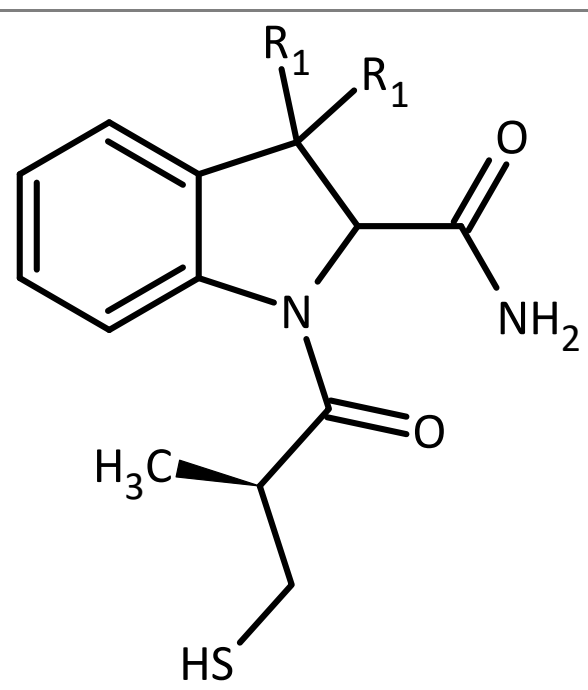	6a (17.9)	Moderate	↑	Thiol (ZBG); Stereochemistry; Hydrophobic cap; Anchoring group effects; Pyrrolidine ring replacement
				6b (4.1)	High	↑↑	
				6c (8.4)	High	↑↑	
				6d (3.5)	High	↑↑	

Group	Ref.	Compound	Structure	IC₅₀, µM	Activity	Activity compared to D-captopril	Key SAR features
A	[42]	**7a-d**	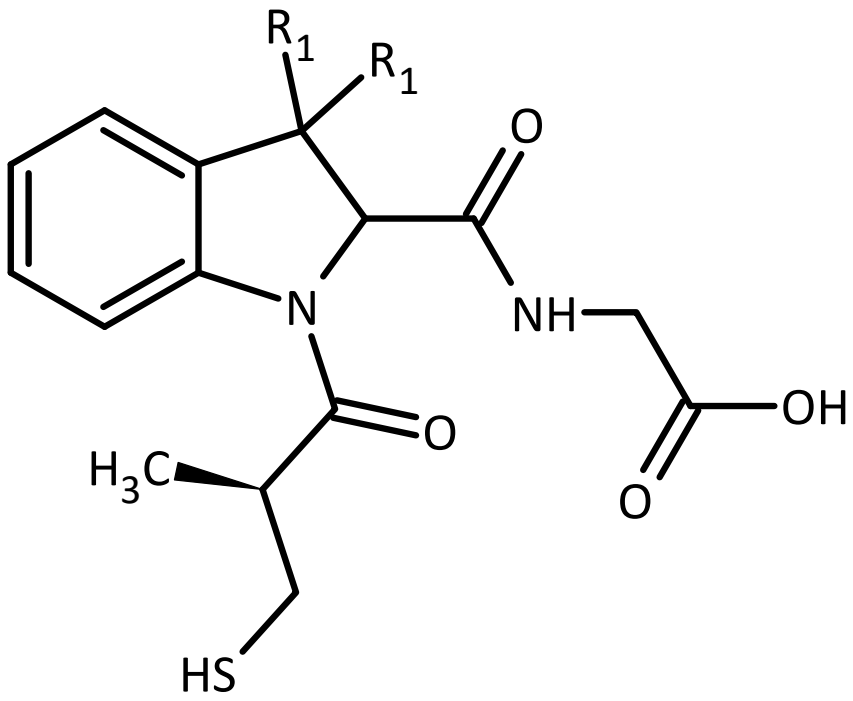	7a(40)<br>7b(8)<br>7c(41)<br>7d(10)	Weak<br>High<br>Weak<br>Moderate	↓<br>↑↑<br>↓<br>↑	Thiol (ZBG); Stereochemistry; Hydrophobic cap; Pyrrolidine ring replacement
A	[42]	**14b-c**	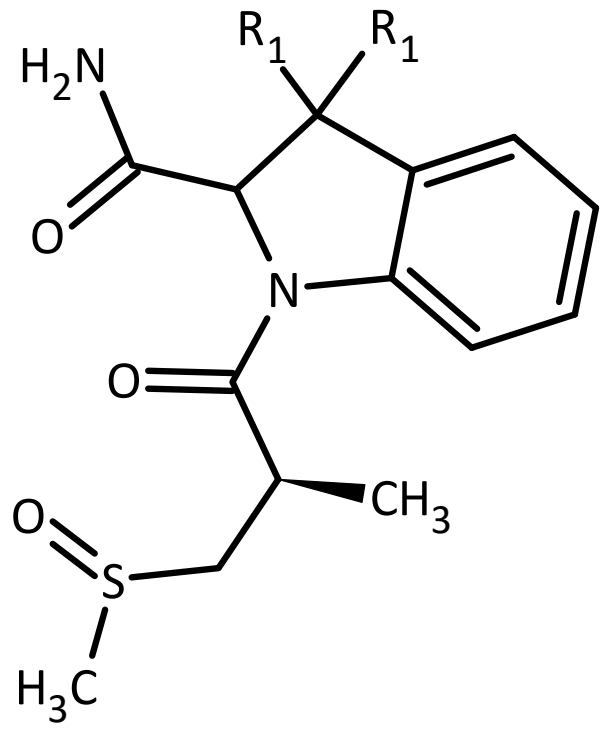	Not determined	Inactive	↓	Thiol masking
A	[42]	**(S,R)-6b**	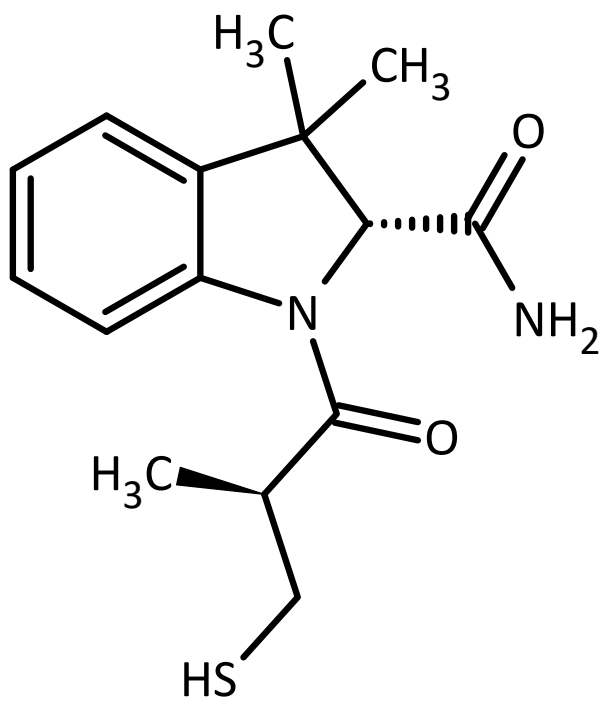	4.1	High	↑↑	Thiol (ZBG); Stereochemistry; Hydrophobic cap; Pyrrolidine ring replacement
A	[42]	**(S,S)-6a**	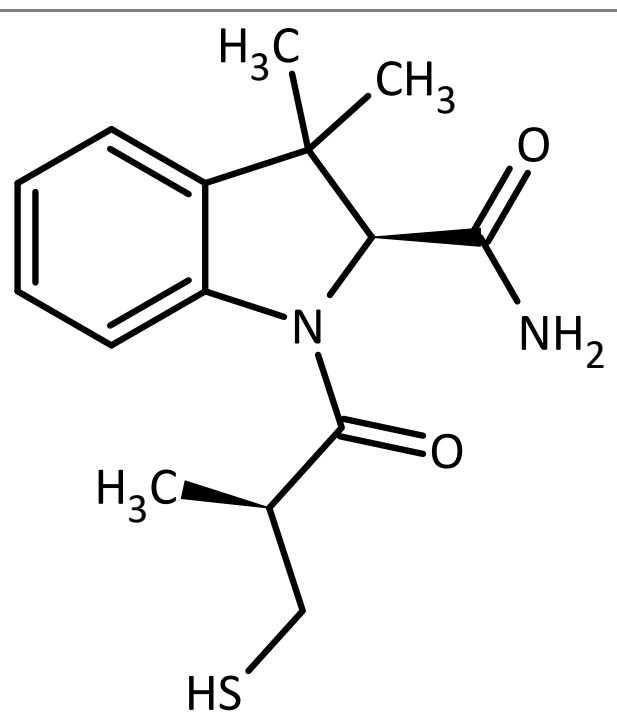	17.9	Moderate	↑	Thiol (ZBG); Stereochemistry; Hydrophobic cap; Pyrrolidine ring replacement
A	[42]	**(S,R)-7b**	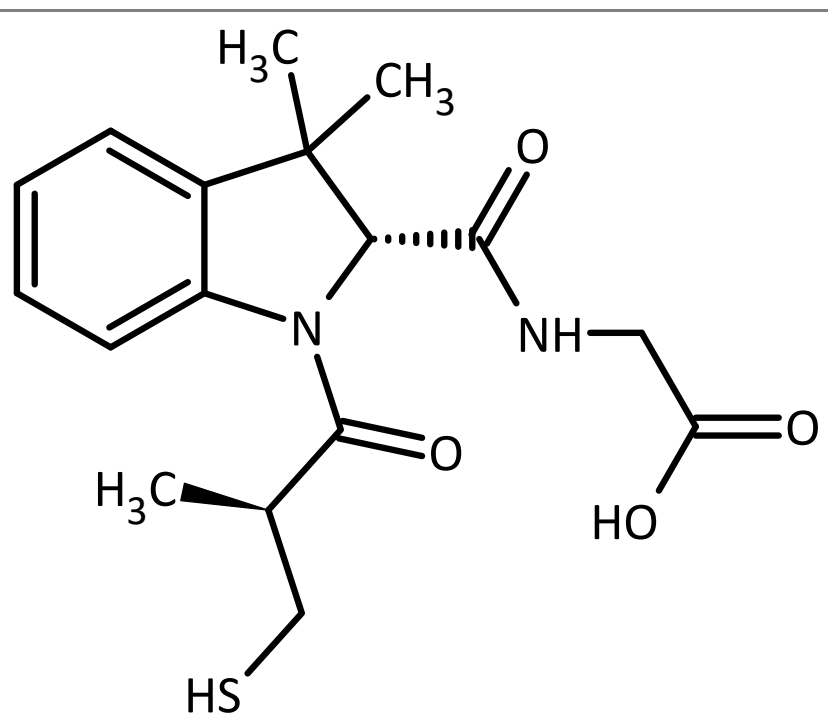	8	High	↑↑	Thiol (ZBG); Stereochemistry; Hydrophobic cap; Pyrrolidine ring replacement

CC BY

Group	Ref.	Compound	Structure	IC₅₀, µM	Activity	Activity compared to D-captopril	Key SAR features
B	[38]	**3**	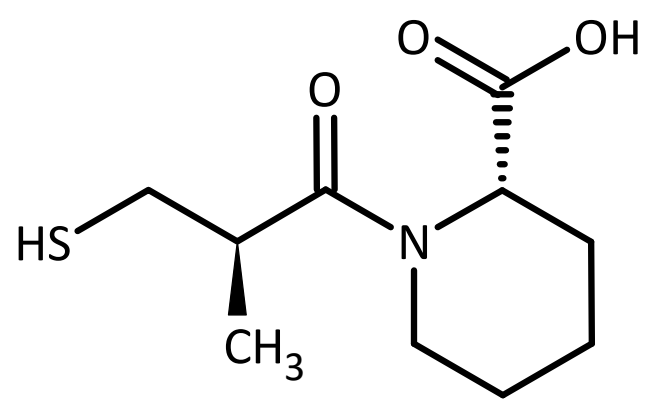	6.8	High	↑↑	Thiol (ZBG); Stereochemistry; Hydrophobic cap; Anchoring group effects (ortho); Pyrrolidine ring expansion
B	[38]	**4**	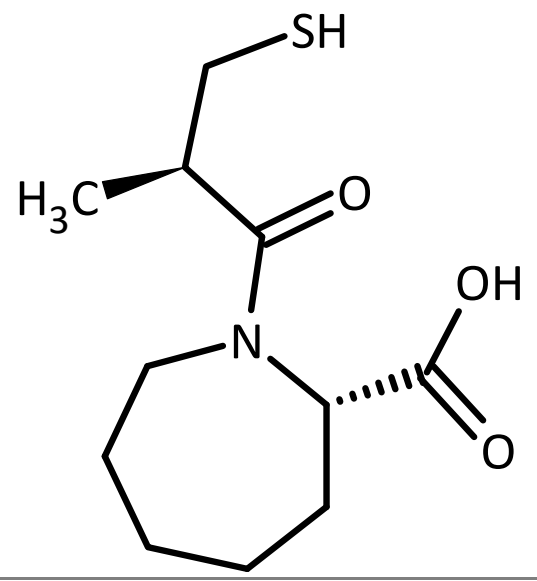	20.4	Weak	≈	Thiol (ZBG); Stereochemistry; Hydrophobic cap; Pyrrolidine ring expansion
B	[38]	**5**	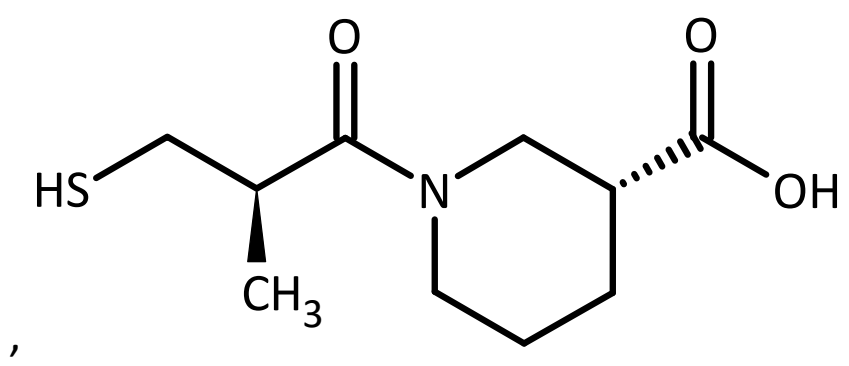	28.4	Weak	↓	Thiol (ZBG); Stereochemistry; Hydrophobic cap; Anchoring group effects (Meta position); Pyrrolidine ring expansion
B	[38]	**6**	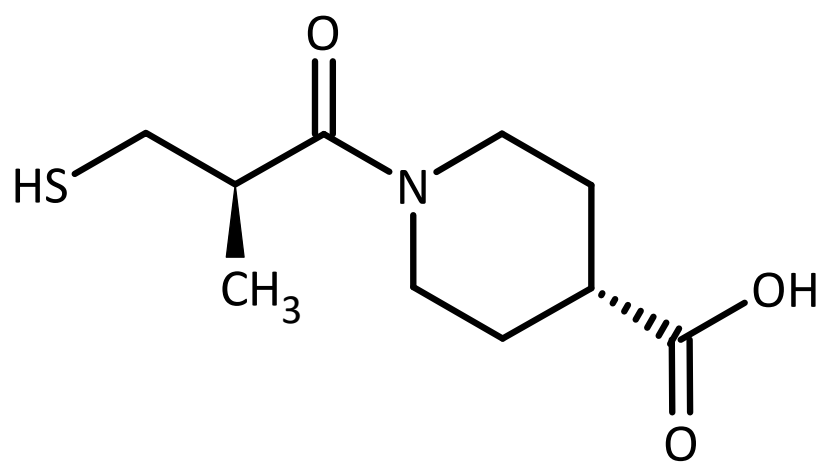	4.9	High	↑↑	Thiol (ZBG); Stereochemistry; Hydrophobic cap;Anchoring group effects (Para) position ; Pyrrolidine ring expansion
B	[38]	**Comp 7**	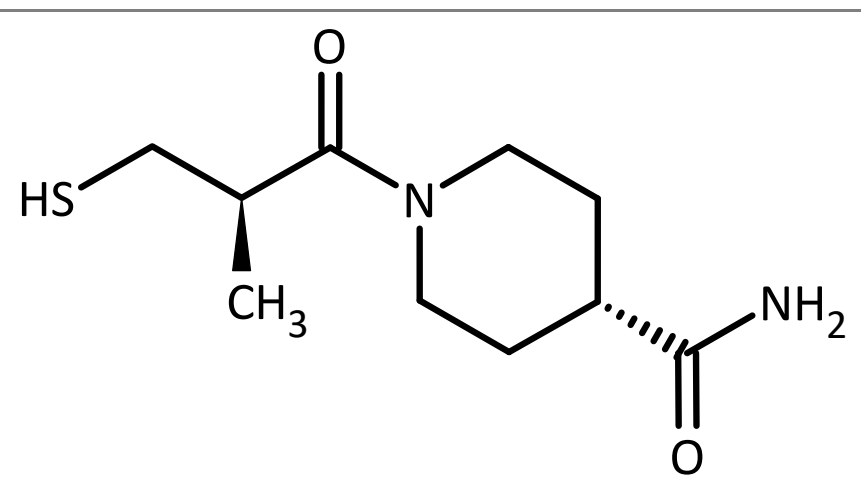	11.1	Moderate	↑	Thiol (ZBG); Stereochemistry; Hydrophobic cap fit; Anchoring group effects (Para); Pyrroli-dine ring expansion
B	[38]	**8**	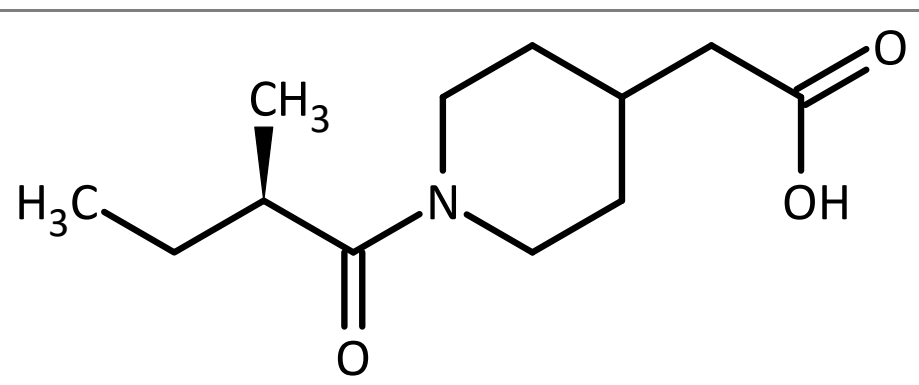	19.8	Moderate	≈	Thiol (ZBG); Stereochemistry; Hydrophobic cap; Pyrrolidine ring expansion Anchoring group effects (meta);
B	[38]	**10**	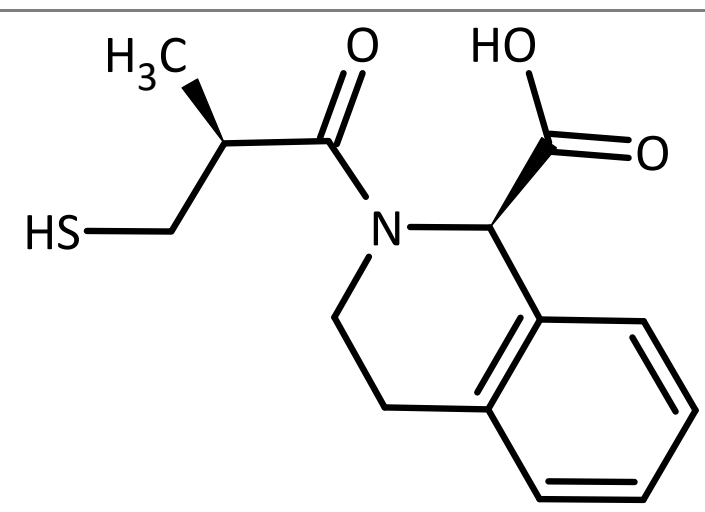	>300	Inactive	↓↓	Excessive enlargement of ring with poor orientation
B	[38]	**11**	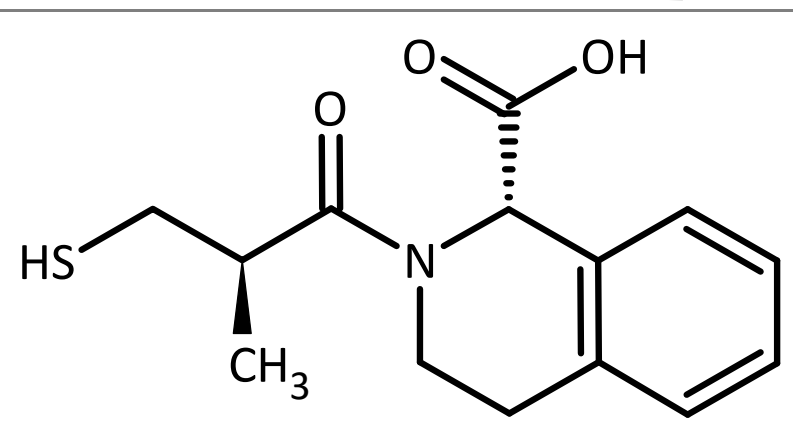	4.6	High	↑↑	Thiol (ZBG); Stereochemistry; Hydrphobic cap Fluorination effect (α position); Pyrrolidine ring expansion

Group	Ref.	Compound	Structure	IC_{50}, μM	Activity	Activity compared to D-captopril	Key SAR features
B	[43]	**(2R,2'S)-5αC**	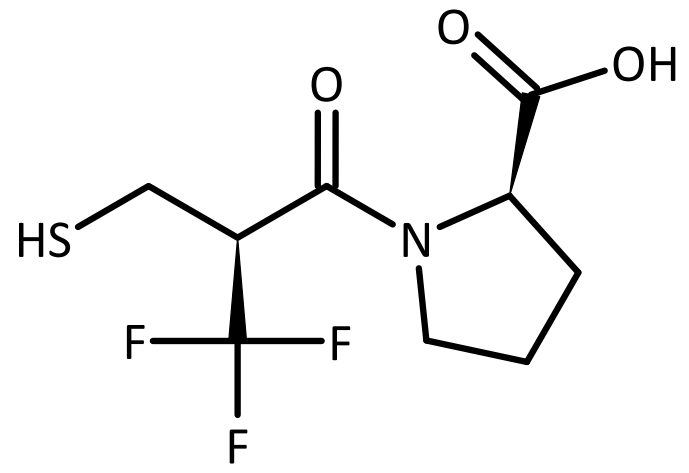	4.5	High	↑↑	Thiol (ZBG); Stereochemistry; Fluorination effect (α position);
B	[43]	**(2R,2'R)-5αC**	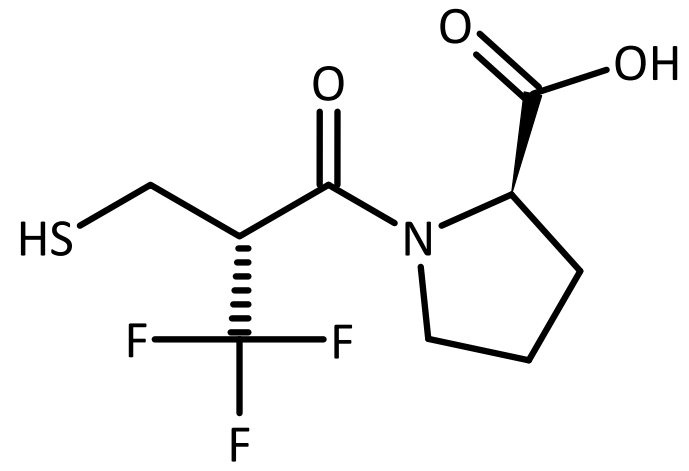	0.3	Very high	↑↑↑	Thiol (ZBG); Stereochemistry; Fluorination effect (α position)
B	[43]	**5αE**	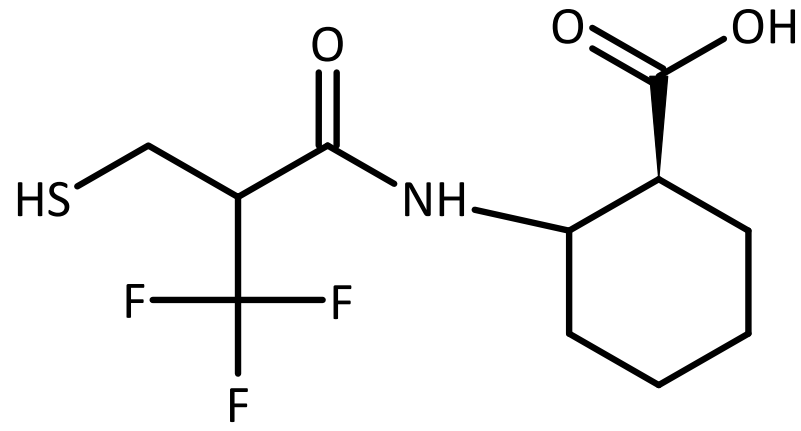	3.2	High	↑↑	Fluorination effect (α position); Pyrrolidine ring expansion
B	[43]	**5βE**	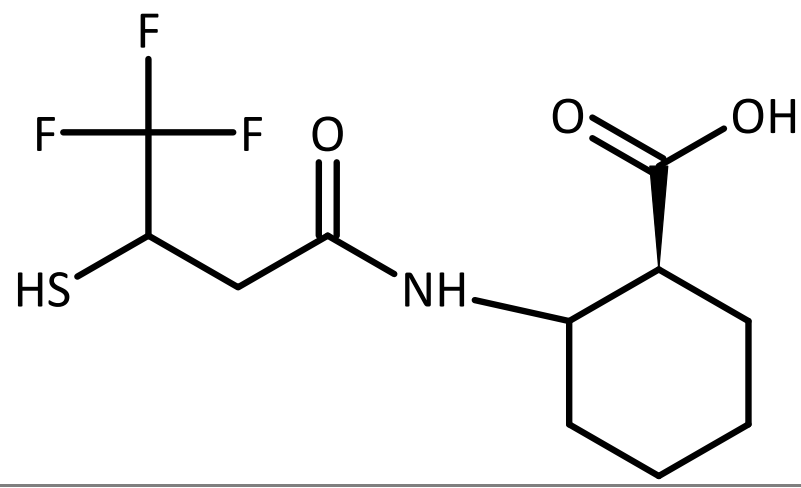	145	Weak	↓	Fluorination effect (β position)
B	[43]	**5αF**	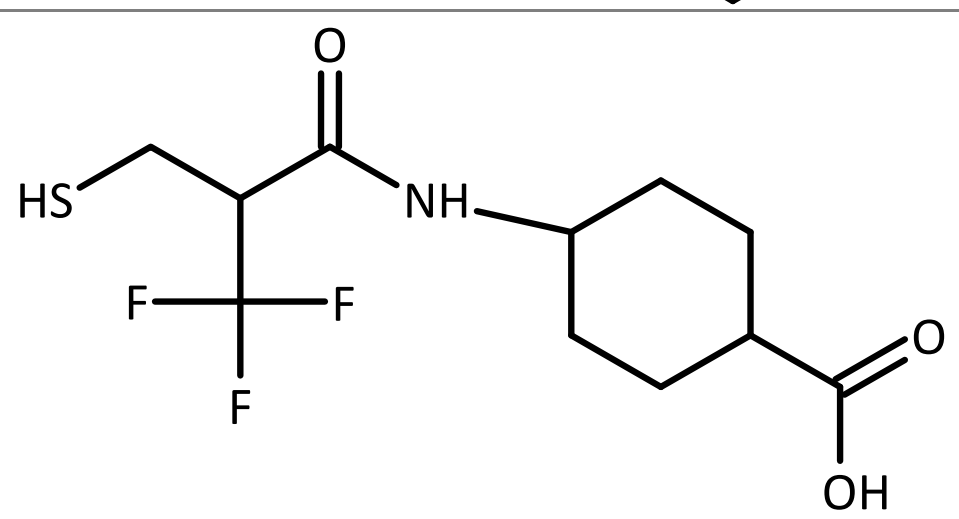	2.2	High	↑↑	Thiol (ZBG); Fluorination effect(α position); Pyrrolidine ring expansion; Anchoring group effects (Para)
C	[40]	**9**	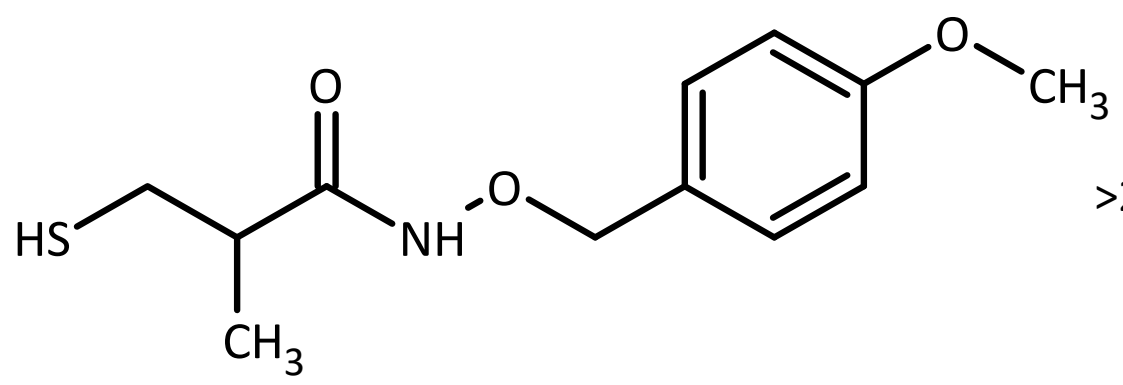	>200	Inactive	↓↓	Anchoring group effects: esterification
C	[40]	**21**	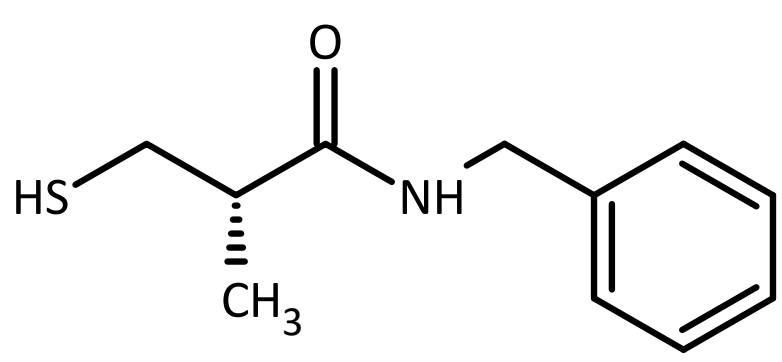	5	High	↑↑	Thiol (ZBG); Stereochemistry Hydrophobic cap; Anchoring group effects; Pyrrolidine ring replacement
C	[40]	**22**	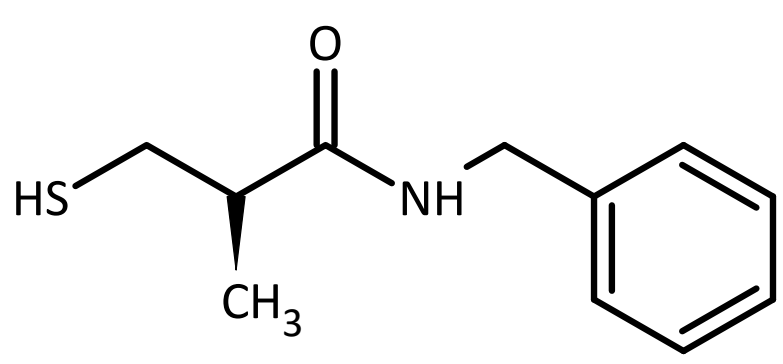	1.5	High	↑↑	Thiol (ZBG); Stereochemistry Hydrophobic cap; Anchoring group effects; Pyrrolidine ring replacement;

CC BY

Group	Ref.	Compound	Structure	IC_{50}, µM	Activity	Activity compared to D-captopril	Key SAR features
C	[40]	**23**	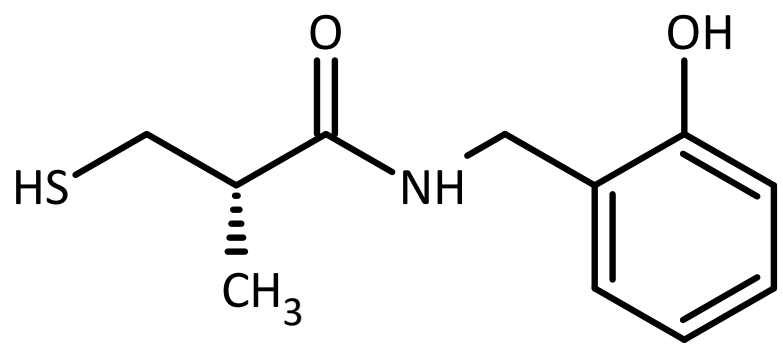	~5	High	↑↑	Thiol (ZBG); Hydrophobic cap Anchoring group effects; Pyrrolidine ring replacement
C	[40]	**26**	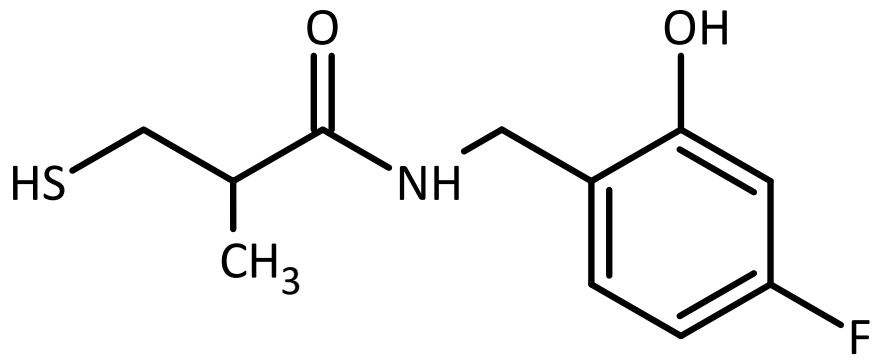	>200	Inactive	↓↓	Fluorination effect; Pyrrolidine ring replacement
C	[40]	**31**	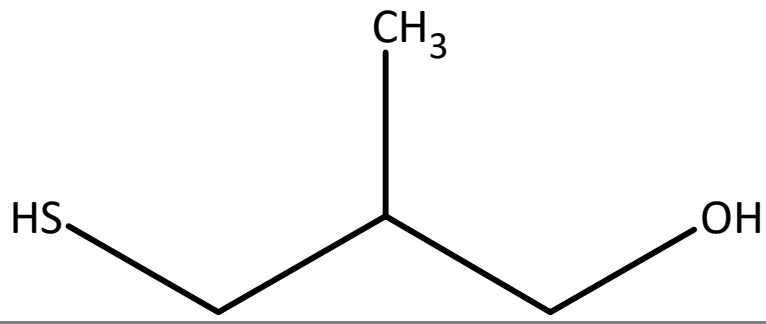	15	Moderate	↑	Thiol (ZBG);Simplification
C	[40]	**32**	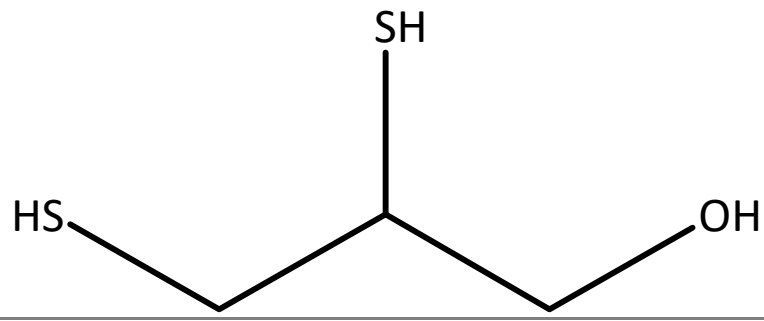	10.4	Moderate	↑↑	Thiol (ZBG);Simplification
C	[39]	D-captopril	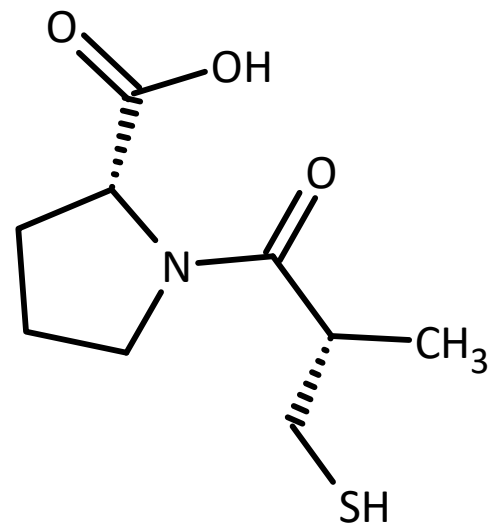	20.1	Weak	Reference compound	Thiol (ZBG); Stereochemistry; Anchoring group orientation
C	[39]	L-captopril	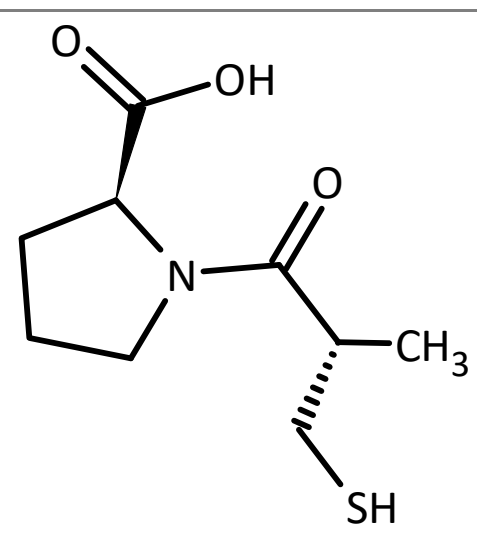	157.4	Weak	↓	Thiol (ZBG); Stereochemistry; Anchoring group orientation
C	[39]	epi-D-captopril	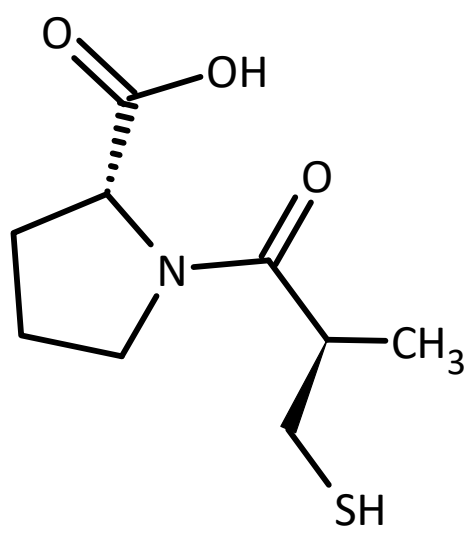	64.6	Weak	↓	Thiol (ZBG); Stereochemistry; Anchoring group orientation
C	[39]	epi-L-captopril	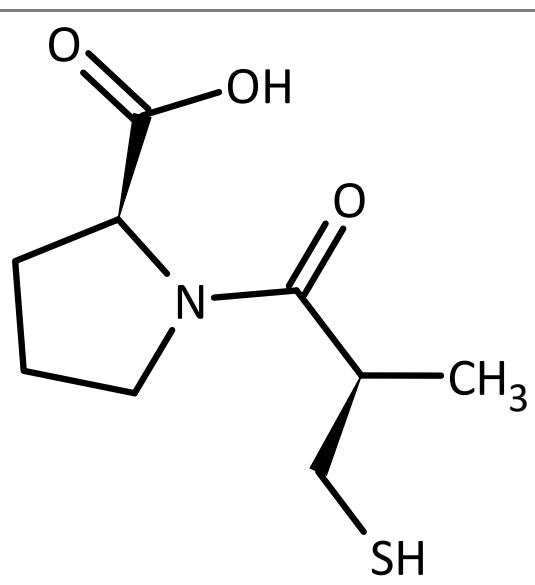	>500	Inactive	↓↓	Thiol (ZBG); Stereochemistry; Anchoring group orientation

Activity: very high: <1 µM, high: 1 to 10 µM, moderate: 10 to 20 µM, weak: 20 to 200 µM, inactive: >200 µM
