# Supplementary material for: Structure-activity relationship of captopril derivatives as New Delhi metallo beta-lactamase 1 inhibitors
Source: ADMET DMPK. 2026 May 24;14:3304. doi: 10.5599/admet.3304 (PMC13499674; doi:10.5599/admet.3304)
Supplement: Supplementary file 2 [file ADMET-14-3304-S1.docx]

ADMET & DMPK **14** (2026) S3304

**Open Access : ISSN : 1848-7718**
[**http://www.pub.iapchem.org/ojs/index.php/admet**](http://www.pub.iapchem.org/ojs/index.php/admet)

Supplementary material to

**Structure-activity relationship of captopril derivatives as
New Delhi metallo beta-lactamase 1 inhibitors**

Ikhlas Jarrar^<https://orcid.org/0000-0002-2232-5928>^

*Department of Pharmaceutical Chemistry, Faculty of Pharmacy, Arab American University, Jenin, Palestine*

ADMET & DMPK **14** (2026) 3304; <https://doi.org/10.5599/admet.3304>

**Table S1.** Captopril derived NDM-1 inhibitors reported in the review, including compound names (Comp.), structures, inhibitory potency, effect on activity compared to D-captopril, and key structure-activity relationship (SAR) features

| Group | Ref. | Compound | Structure | IC₅₀, µM | Activity | | Activity compared to D-captopril | Key SAR features |
| --- | --- | --- | --- | --- | --- | --- | --- | --- |
| A | [41] | **14a** |  | 0.10 | Very high | | ↑↑↑ | Thiol (ZBG); Hydrophobic cap; Anchoring group effects; Pyrrolidine ring replacement |
| A | [41] | **14m-1 (S,S)** |  | 0.48 | Very high | | ↑↑↑ | Thiol (ZBG); Stereochemistry;  Hydrophobic cap; Anchoring group effects; Pyrrolidine ring replacement |
| A | [41] | **14m-2 (R,S)** |  | 0.17 | Very high | | ↑↑↑ | Thiol (ZBG); Stereochemistry;  Hydrophobic cap; Anchoring group effects; Pyrrolidine ring replacement |
| A | [41] | **14q** |  | 3.90 | High | | ↑↑ | Thiol (ZBG);  Fluorination; Pyrrolidine ring replacement |
| A | [42] | **6a-d** |  | 6a (17.9) | Moderate | | ↑ | Thiol (ZBG); Stereochemistry;  Hydrophobic cap; Anchoring group effects; Pyrrolidine ring replacement |
|  |  |  |  | 6b (4.1) | High | | ↑↑ |  |
|  |  |  |  | 6c (8.4) | High | | ↑↑ |  |
|  |  |  |  | 6d (3.5) | High | | ↑↑ |  |
| A | [42] | **7a-d** |  | 7a(40) | Weak | | ↓ | Thiol (ZBG); Stereochemistry;  Hydrophobic cap; Pyrrolidine ring replacement |
|  |  |  |  | 7b(8) | High | | ↑↑ |  |
|  |  |  |  | 7c(41) | Weak | | ↓ |  |
|  |  |  |  | 7d(10) | Moderate | | ↑ |  |
| A | [42] | **14b-c** |  | Not determined | Inactive | | ↓ | Thiol masking |
| A | [42] | **(S,R)-6b** |  | 4.1 | High | | ↑↑ | Thiol (ZBG); Stereochemistry;  Hydrophobic cap; Pyrrolidine ring replacement |
| A | [42] | **(S,S)-6a** |  | 17.9 | Moderate | | ↑ | Thiol (ZBG); Stereochemistry;  Hydrophobic cap; Pyrrolidine ring replacement |
| A | [42] | **(S,R)-7b** |  | 8 | High | | ↑↑ | Thiol (ZBG); Stereochemistry;  Hydrophobic cap; Pyrrolidine ring replacement |
| B | [38] | **3** |  | 6.8 | High | | ↑↑ | Thiol (ZBG); Stereochemistry;  Hydrophobic cap; Anchoring group effects (ortho); Pyrrolidine ring expansion |
| B | [38] | **4** |  | 20.4 | Weak | | ≈ | Thiol (ZBG); Stereochemistry;  Hydrophobic cap; Pyrrolidine ring expansion |
| B | [38] | **5** | ,  | 28.4 | Weak | | ↓ | Thiol (ZBG); Stereochemistry;  Hydrophobic cap; Anchoring group effects (Meta position); Pyrrolidine ring expansion |
| B | [38] | **6** |  | 4.9 | High | | ↑↑ | Thiol (ZBG); Stereochemistry;  Hydrophobic cap;Anchoring group effects (Para) position ; Pyrrolidine ring expansion |
| B | [38] | **Comp 7** |  | 11.1 | Moderate | | ↑ | Thiol (ZBG); Stereochemistry;  Hydrophobic cap fit; Anchoring group effects (Para); Pyrroli­dine ring expansion |
| B | [38] | **8** |  | 19.8 | Moderate | | ≈ | Thiol (ZBG); Stereochemistry;  Hydrophobic cap; Pyrrolidine ring expansion  Anchoring group effects (meta); |
| B | [38] | **10** |  | >300 | Inactive | | ↓↓ | Excessive enlargement of ring with poor orientation |
| B | [38] | **11** |  | 4.6 | High | | ↑↑ | Thiol (ZBG); Stereochemistry;  Hydrphobic cap Fluorination effect (α position); Pyrrolidine ring expansion |
| B | [43] | **(2R,2′S)-5αC** |  | 4.5 | High | | ↑↑ | Thiol (ZBG); Stereochemistry; Fluorination effect  (α position); |
| B | [43] | **(2R,2′R)-5αC** |  | 0.3 | Very high | | ↑↑↑ | Thiol (ZBG); Stereochemistry; Fluorination effect  (α position) |
| B | [43] | **5αE** |  | 3.2 | High | | ↑↑ | Fluorination effect  (α position); Pyrrolidine ring expansion |
| B | [43] | **5βE** |  | 145 | Weak | ↓ | | Fluorination effect  (β position) |
| B | [43] | **5αF** |  | 2.2 | High | | ↑↑ | Thiol (ZBG); Fluorination effect(α position); Pyrrolidine ring expansion; Anchoring group effects (Para) |
| C | [40] | **9** |  | >200 | Inactive | | ↓↓ | Anchoring group effects: esterification |
| C | [40] | **21** |  | 5 | High | | ↑↑ | Thiol (ZBG); Stereochemistry Hydrophobic cap; Anchoring group effects; Pyrrolidine ring replacement |
| C | [40] | **22** |  | 1.5 | High | | ↑↑ | Thiol (ZBG); Stereochemistry Hydrophobic cap; Anchoring group effects; Pyrrolidine ring replacement; |
| C | [40] | **23** |  | ~5 | High | | ↑↑ | Thiol (ZBG); Hydrophobic cap Anchoring group effects; Pyrrolidine ring replacement |
| C | [40] | **26** |  | >200 | Inactive | | ↓↓ | Fluorination effect; Pyrrolidine ring replacement |
| C | [40] | **31** |  | 15 | Moderate | | ↑ | Thiol (ZBG);Simplification |
| C | [40] | **32** |  | 10.4 | Moderate | | ↑↑ | Thiol (ZBG);Simplification |
| C | [39] | D-captopril |  | 20.1 | Weak | | Reference compound | Thiol (ZBG); Stereochemistry; Anchoring group orientation |
| C | [39] | L-captopril |  | 157.4 | Weak | | ↓ | Thiol (ZBG); Stereochemistry; Anchoring group orientation |
| C | [39] | epi-D-cap­to­pril |  | 64.6 | Weak | | ↓ | Thiol (ZBG); Stereochemistry; Anchoring group orientation |
| C | [39] | epi-L-cap­to­pril |  | >500 | Inactive | | ↓↓ | Thiol (ZBG); Stereochemistry; Anchoring group orientation |

Activity: very high: <1 µM, high: 1 to 10 µM, moderate: 10 to 20 µM, weak: 20 to 200 µM, inactive: >200 µM
